# Reliability, validity, and screening performance of the Chinese version of the McLean Screening Instrument for Borderline Personality Disorder in a psychiatric clinical sample

**DOI:** 10.3389/fpsyt.2026.1865970

**Published:** 2026-06-24

**Authors:** Yizhong Shen, Danyu Li, Zhi Zeng, Lei Ye, Lanlan Wang, Jingju Quan

**Affiliations:** 1 Department of Psychiatry, The Third People’s Hospital of Zhuhai, Zhuhai, China; 2 Department of Child Mental Health, Zhuhai Maternity and Child Health Hospital, Zhuhai, China; 3 Department of Psychology, Shanghai General Hospital, Shanghai, China

**Keywords:** borderline personality disorder, Chinese version, McLean Screening Instrument for Borderline Personality Disorder, psychiatric clinical sample, reliability, screening performance, theory-driven, validity

## Abstract

**Introduction:**

Evidence regarding the psychometric properties of the Chinese version of the McLean Screening Instrument for Borderline Personality Disorder (MSI-BPD) in psychiatric clinical populations remains limited. This study evaluated the reliability, validity, and screening performance of the Chinese MSI-BPD in a psychiatric clinical sample and examined a theory-driven four-factor model.

**Methods:**

The previously translated Chinese MSI-BPD was reviewed for comprehensibility and clinical appropriateness through expert review, cognitive debriefing, and pilot testing. In this cross-sectional study, 375 outpatients and inpatients from a tertiary psychiatric hospital in Zhuhai, China, completed the Chinese MSI-BPD, with the Revised Diagnostic Interview for Borderlines (DIB-R) used as the reference standard. Confirmatory factor analysis was conducted using the weighted least squares mean- and variance-adjusted estimator. Five CFA models were compared: a one-factor model, a classical four-factor model, a literature-derived modified four-factor model, the theory-driven four-factor model, and a higher-order model. Reliability, criterion-related validity, and screening performance were evaluated.

**Results:**

The theory-driven four-factor model showed the most favorable fit indices among the admissible models (χ²/df = 1.409, CFI = 0.995, TLI = 0.992, RMSEA = 0.033, SRMR = 0.043). The higher-order model also showed good fit, supporting a general BPD factor underlying the four symptom domains. In the theory-driven model, standardized factor loadings ranged from 0.622 to 0.911. The Chinese MSI-BPD total score was strongly correlated with the DIB-R total score (r = 0.829, p < 0.01). McDonald’s omega, Guttman split-half reliability, and test–retest reliability for the total scale were 0.884, 0.894, and 0.851, respectively. The Youden-based optimal cutoff was 6.5, corresponding to a clinically practical threshold of ≥7 in the present sample, with an AUC of 0.884, sensitivity of 88.83%, specificity of 71.91%, and Cohen’s kappa of 0.612.

**Conclusions:**

The Chinese MSI-BPD total score demonstrated good reliability, criterion-related validity, and screening performance in this psychiatric clinical sample. The theory-driven four-factor model may provide a clinically useful but provisional interpretive framework; however, the total score should remain the primary screening index. The instrument may serve as a brief preliminary screening tool for BPD in tertiary psychiatric clinical settings in China, but not in general psychiatric or community populations without dedicated validation.

## 1 Introduction

Borderline personality disorder (BPD) is a severe mental disorder characterized by affective dysregulation, cognitive disturbance, interpersonal instability, and impulsive behavior, with marked impairment in psychosocial functioning (1) and an increased risk of self-harm and suicide (2–4). Early identification of BPD remains a major clinical challenge. Because its symptoms often overlap with those of bipolar disorder, schizophrenia-spectrum disorders, and other psychiatric conditions, insufficient theoretical understanding and limited diagnostic awareness may contribute to misdiagnosis or under-recognition (5–7). Early identification and timely intervention are clinically important for improving long-term outcomes (8).

Recognition and research on BPD remain relatively limited in psychiatric practice in China (9). Earlier domestic diagnostic systems did not formally include BPD (10, 11), and the high comorbidity between BPD and other psychiatric disorders has further complicated recognition in routine practice (12, 13). Therefore, improving screening efficiency through appropriate instruments is clearly of practical importance. At present, however, validated tools for BPD assessment in Chinese psychiatric clinical settings remain limited (10, 14). Compared with lengthy diagnostic interviews, such as the Structured Clinical Interview for DSM-IV Axis II Disorders and the International Personality Disorder Examination (15, 16), self-report instruments are more feasible for initial screening in busy outpatient and inpatient settings (17).

The McLean Screening Instrument for Borderline Personality Disorder (MSI-BPD) is a brief self-report screening tool developed on the basis of Diagnostic and Statistical Manual of Mental Disorders, Fourth Edition (DSM-IV) criteria. It contains 10 items and has shown good psychometric performance in previous studies conducted on Western samples (18–20). However, most prior evidence has come from North American or European populations, and the tool’s applicability to psychiatric clinical samples in mainland China remains to be established. Given that symptom expression and clinical recognition may be influenced by cultural context (10, 21), systematic evaluation of the MSI-BPD in Chinese psychiatric clinical samples is warranted.

Although the MSI-BPD has been widely used in Western samples, its internal structure may vary across cultural contexts and sample types (22–24). It is therefore important to further examine its structure in Chinese psychiatric clinical samples. BPD is not a unitary symptom cluster but rather comprises several interrelated yet relatively distinct psychopathological domains. Previous studies have generally suggested that the theoretical structure of BPD can be conceptualized in terms of four major dimensions (25, 26) (1): affective disturbance, including affective instability, inappropriate anger, and chronic feelings of emptiness (2); cognitive disturbance, including identity disturbance, dissociative symptoms, and nondelusional suspiciousness (3); impulsivity, including impulsive behaviors and self-harm or suicidal behaviors; and (4) unstable relationships, including chaotic intimate relationships and fear of abandonment. This framework provides an important theoretical basis for understanding the organization of BPD symptoms. Studies on Asian individuals, particularly Chinese individuals (22, 27), have also suggested that the theoretical structure of BPD can still be broadly conceptualized in terms of four core dimensions described above: affective dysregulation, impulsivity, cognitive disturbance, and interpersonal dysfunction.

On the basis of this theoretical framework and the clinical meanings of individual MSI-BPD items, we specified a four-factor model *a priori*. In this model, MSI4 (marked mood instability) and MSI5 (frequent anger or sarcasm) were assigned to the affective factor; MSI2 (self-harm/suicidal behavior) and MSI3 (multiple impulsive behaviors) were assigned to the impulsive factor; MSI1 (repeated deterioration of close relationships) and MSI10 (desperate efforts to avoid abandonment) were assigned to the interpersonal factor; and MSI6 (distrust of others), MSI7 (feeling unreal), MSI8 (chronic feelings of emptiness), and MSI9 (identity disturbance) were assigned to the cognitive factor. Notably, although chronic emptiness is often classified under affective dysregulation in classical theoretical models of BPD (25, 26), the latent assignment of a specific item within a given screening instrument should also be judged according to item semantics and its organizational relationship with other symptom contents. Chronic emptiness is intrinsically cross-domain: it may manifest both as a lack of emotional experience and as a disturbance in self-continuity and internal representation (28–31). In the context of Chinese culture, in which self-awareness is often more deeply embedded in significant others (32), social roles, and situational evaluation, “inner emptiness” may be experienced not only as affective deprivation but also as a reduced sense of inner support, weakened subjectivity, and unstable self-identity. Within the MSI-BPD, MSI8 appears to reflect not only mood fluctuations but also a persistent sense of internal hollowness, deficient self-representation, and a diminished sense of self-presence. In terms of clinical meaning, it is more closely aligned with MSI7 (feelings of unreality) and MSI9 (identity instability) (33), both of which involve abnormalities in self-awareness and instability of internal cognitive representation. Therefore, while retaining the overarching four-domain framework of BPD—affective, cognitive, impulsive, and interpersonal—we grouped MSI8 together with MSI6, MSI7, and MSI9 under the cognitive factor and tested the applicability of this theory-driven specification in a Chinese psychiatric clinical sample.

The Chinese version of the MSI-BPD was first translated and examined in a college student sample by Wang, Liang, and Zhong (2008) (34). Subsequently, Chen et al. (2011) (35) conducted an early domestic psychometric study of the MSI-BPD in Chinese psychiatric clinical samples. Their findings showed that the scale had acceptable internal consistency and that the modified four-factor model demonstrated the best fit among the tested models. In this model, MSI4 and MSI5 were assigned to the affective factor, MSI2 and MSI3 to the impulsive factor, MSI7, MSI8, and MSI9 to the cognitive factor, and MSI1, MSI6, and MSI10 to the interpersonal factor. This early study provided important preliminary evidence for the application of the MSI-BPD in Chinese psychiatric clinical samples.

Nevertheless, several issues remain to be further addressed. First, although Chen et al. reported internal consistency evidence and examined factor structure, test–retest reliability, split-half reliability, AVE/CR-based construct validity, and ROC-derived screening-performance indices were not systematically examined. Second, that study did not establish a clinically practical screening cutoff against a semi-structured diagnostic reference standard. Third, the fit and interpretability of alternative theoretically plausible CFA models, including a theory-driven item-domain allocation based on core BPD psychopathological domains, require further evaluation in contemporary psychiatric clinical samples. Therefore, the primary aim of the present study was to examine a more comprehensive set of reliability, validity, and ROC-derived screening-performance indices of the Chinese MSI-BPD in a psychiatric clinical sample, using the DIB-R as the semi-structured reference standard, and to determine a clinically practical screening cutoff. The secondary aim was to compare several plausible CFA models, including the modified four-factor model reported by Chen et al. and the present theory-driven four-factor model, thereby clarifying the incremental psychometric evidence for the use of the Chinese MSI-BPD in tertiary psychiatric clinical settings in China.

## 2 Materials and methods

### 2.1 Study sites and participants

This study was conducted in a tertiary psychiatric hospital with 800 beds in Zhuhai, Guangdong Province, China. Using a cross-sectional design, we recruited outpatient and inpatient participants between 1 November 2024 and 30 June 2025. Participants were recruited using convenience sampling among clinically available eligible outpatients and inpatients who agreed to participate, rather than through population-based sampling. Therefore, the sample should be understood as a tertiary psychiatric clinical sample rather than a representative general psychiatric outpatient or inpatient population. Because the primary aim was to evaluate the Chinese MSI-BPD as a brief screening instrument in routine tertiary psychiatric clinical settings, both adolescent and adult patients who met the eligibility criteria were included in the main analysis. To address potential developmental differences, age-stratified reliability and ROC analyses were conducted as exploratory supplementary analyses. The inclusion criteria were as follows: no severe physical illness, adequate reading comprehension and understanding to complete the assessment independently, and voluntary provision of informed consent. The exclusion criteria were being in an acute episode of severe psychiatric illness; having intellectual disability; having chronic severe deterioration, dementia, or other conditions associated with substantial cognitive impairment; having organic mental disorders; having personality changes secondary to traumatic brain injury; having severe language or reading comprehension difficulties; and having severe physical conditions that precluded questionnaire completion.

A total of 387 questionnaires were distributed and returned, 375 of which were considered valid and included in the final analyses. Twelve questionnaires were excluded because they contained incomplete responses to key assessment items or invalid response patterns. For the 375 valid questionnaires included in the analyses, there were no missing item-level data for the Chinese MSI-BPD items or the main DIB-R variables used in the present analyses; therefore, no item-level imputation was performed. The sample size for this study was primarily determined according to commonly used empirical criteria for confirmatory factor analysis (CFA). The MSI-BPD consists of 10 items. On the basis of the general rule in psychometric research that the sample size should be at least 10–20 times the number of items, a minimum of 100–200 participants was needed. Given the relatively high sample size requirements for CFA, a total sample size of no fewer than 300 is generally recommended. The final sample of 375 participants was therefore considered sufficient for psychometric evaluation and screening performance analysis. The study was approved by the Ethics Committee of the Third People’s Hospital of Zhuhai. After being informed in person about the study content and purpose, all participants were provided with an electronic informed consent form. For participants younger than 18 years, informed consent was obtained from their legal guardians, and assent was obtained from the participants where appropriate.

### 2.2 Clinical review and cognitive debriefing of the Chinese MSI-BPD

The Chinese version of the MSI-BPD used in this study was based on the translation previously reported by Wang, Liang, and Zhong (2008) (34). Because that version had been evaluated primarily in college students, we further reviewed its comprehensibility and clinical appropriateness for use in psychiatric patients before formal data collection.

First, an expert panel consisting of three senior psychiatrists and one clinical psychologist reviewed the existing Chinese items with reference to the original English version. The panel focused on semantic clarity, idiomatic appropriateness, and clinical relevance in psychiatric settings.

Second, cognitive debriefing interviews were conducted with 12 psychiatric outpatients and inpatients (6 with mood disorders and 6 with anxiety or personality disorders). Participants were asked to paraphrase each item and explain what it meant to them. Expressions that were difficult to understand or potentially ambiguous were identified and discussed by the panel.

Before formal data collection, the Chinese version was piloted in 20 psychiatric patients to confirm acceptability and comprehension. The final Chinese version retained the original item wording, number of items, response format, and scoring method.

### 2.3 Measures

#### 2.3.1 Demographic questionnaire

Demographic information included sex, age, educational level, marital status, whether the participant had grown up in a reconstituted family following parental divorce, and whether the participant had siblings.

#### 2.3.2 McLean Screening Instrument for Borderline Personality Disorder

The MSI-BPD is a self-report screening instrument for BPD developed by Zanarini and colleagues in 2003 on the basis of the Diagnostic Interview for DSM-IV Personality Disorders. The scale contains 10 items. Each item is answered “yes” (1 point) or “no” (0 points), with higher total scores indicating more pronounced borderline personality pathology. The 10 items correspond to the 9 DSM-IV BPD criteria, with the first 8 criteria represented by one item each and the ninth criterion (paranoid ideation/dissociation) represented by two items. Previous studies on Western samples have demonstrated good reliability and validity (18–20). Prior research has shown that a cutoff score of 7 yields good sensitivity (0.81) and specificity (0.85) for identifying BPD (19, 36). The Chinese version used in this study was based on the translation reported by Wang et al. (2008) (34) and was reviewed for comprehensibility and clinical appropriateness in psychiatric patients, as described in Section 2.2. No formal changes were made to the item wording, number of items, response options, or scoring procedures. In the present study, a theory-driven latent factor structure was specified *a priori* and then examined empirically.

#### 2.3.3 Reference criterion: Revised Diagnostic Interview for Borderlines

The DIB-R, adapted from the original DIB (37), is a semistructured interview designed for the diagnostic assessment of BPD. It contains 127 items across four sections: affect (26 items), cognition (34 items), impulsive action patterns (24 items), and interpersonal relationships (43 items). Ninety-seven first-level items assess feelings, thoughts, and behavior patterns over the previous two years; 22 second-level items summarize the first-level items; and 8 third-level items are used to calculate the final scores on the basis of the second-level items. Previous studies have demonstrated good reliability and validity of the DIB-R (27, 38). At a cutoff score of 8, the DIB-R has a sensitivity of 82% and a specificity of 80% (27, 38).

### 2.4 Data collection procedures

Five attending psychiatrists participated in this study, each of whom was responsible for interviewing and evaluating eligible outpatients and inpatients. Before data collection, all psychiatrists received face-to-face training on the study protocol, ethics and informed consent procedures, DIB-R administration and scoring rules, the clinical theory and diagnostic criteria of BPD, and the handling of ambiguous interview responses. The training was intended to standardize the interview process and reduce variability in DIB-R administration across interviewers.

After presenting to the outpatient clinic or being admitted to the ward, each patient was informed of the study purpose by the evaluating psychiatrist. Patients then accessed the assessment system via smartphone scanning and completed the demographic questionnaire and the Chinese MSI-BPD independently. The initial page of the survey contained the informed consent section. Patients and, where applicable, their guardians who agreed to participate selected the consent option and proceeded to the formal assessment; those who declined exited the system.

After self-report completion, the evaluating psychiatrist conducted a face-to-face DIB-R interview in a relatively quiet independent area. All assessments were completed within 24 hours of the patient’s clinical visit, and the overall assessment process lasted approximately 30–60 minutes. The Chinese MSI-BPD was completed independently by the patients, whereas the DIB-R was administered by trained attending psychiatrists who were blinded to the Chinese MSI-BPD scores. Although interviewer training and blinding procedures were implemented as quality-control measures, formal independent co-rating and calculation of DIB-R inter-rater reliability were not performed in the present study.

In addition, 20 participants were randomly selected from the valid sample and completed the Chinese MSI-BPD again 2–3 weeks later for assessment of test–retest reliability. To examine the comparability of the retest subsample, the retest participants were compared with the full sample on key demographic and clinical variables, including sex, age group, DIB-R status, baseline MSI-BPD total score, and DIB-R total score.

Primary clinical diagnoses were determined by psychiatrists according to the International Classification of Diseases, 10th Revision (ICD-10) diagnostic criteria and clinical information. In this study, disease categories were classified based on the primary diagnosis. When organizing the primary diagnoses, depressive disorders and bipolar affective disorder were listed separately, while cases recorded in the medical records as mood disorders without further specification were classified as “other mood disorders.”

### 2.5 Statistical analysis

IBM SPSS 22.0 was used for descriptive and general statistical analyses, and R (lavaan package) was used for confirmatory factor analysis (CFA) and additional model comparisons. Continuous variables are presented as the means ± standard deviations, and categorical variables are presented as frequencies and percentages. Demographic variables were summarized descriptively to characterize the study sample. Because the items of the Chinese MSI-BPD are dichotomous, CFA was conducted using the weighted least squares mean- and variance-adjusted (WLSMV) estimator. Model fit was evaluated using χ²/df, the Comparative Fit Index (CFI), Tucker–Lewis Index (TLI), root mean square error of approximation (RMSEA) with 90% confidence intervals (CIs), and standardized root mean square residual (SRMR).

On the basis of the four-domain theoretical structure of BPD and the clinical meanings of MSI-BPD items, a theory-driven four-factor model was specified *a priori* as follows: affective factor (MSI4, MSI5), impulsive factor (MSI2, MSI3), cognitive factor (MSI6, MSI7, MSI8, MSI9), and interpersonal factor (MSI1, MSI10). CFA was used to assess structural validity. To evaluate the relative fit and interpretability of this *a priori* theory-driven model, it was compared with four alternative or literature-derived models of the Chinese MSI-BPD. These included a one-factor model in which all 10 items loaded on a general BPD factor, serving as a parsimonious baseline model because the MSI-BPD is primarily used as a total-score screening instrument; a classical four-factor model in which MSI4, MSI5, and MSI8 loaded on the affective factor, MSI2 and MSI3 on the impulsive factor, MSI6, MSI7, and MSI9 on the cognitive factor, and MSI1 and MSI10 on the interpersonal factor; the modified four-factor model reported by Chen et al., in which MSI4 and MSI5 loaded on the affective factor, MSI2 and MSI3 on the impulsive factor, MSI7, MSI8, and MSI9 on the cognitive factor, and MSI1, MSI6, and MSI10 on the interpersonal factor; and a higher-order model based on the theory-driven four-factor structure, in which the four first-order factors loaded onto a general BPD factor. For the theory-driven four-factor model, standardized factor loadings were extracted, and the average variance extracted (AVE) and composite reliability (CR) were calculated to evaluate convergent validity. Discriminant validity was assessed using the Fornell–Larcker criterion by comparing the square root of the AVE for each factor with the interfactor correlations.

Criterion-related validity was examined using Pearson correlations between Chinese MSI-BPD total and dimension scores and DIB-R total and corresponding dimension scores. Reliability analysis included McDonald’s omega, Guttman split-half reliability, and test–retest reliability. For comparison between the test–retest subsample and the full sample, categorical variables were compared using Fisher’s exact tests and continuous variables were compared using Mann–Whitney U tests because of the small size of the retest subsample. Screening performance was evaluated using receiver operating characteristic (ROC) analysis with the DIB-R as the reference standard, and the area under the curve (AUC) was calculated. The optimal cutoff was determined using the maximum Youden index. Sensitivity, specificity, positive predictive value (PPV), negative predictive value (NPV), accuracy, false-positive rate, false-negative rate, and Cohen’s kappa were calculated. Confidence intervals for proportion-based indices were estimated using the Wilson method, and the confidence interval for Cohen’s kappa was estimated using bootstrap resampling with 5,000 repetitions. Stratified reliability and ROC analyses by sex and age group were conducted as exploratory supplementary analyses. For exploratory subgroup reliability analyses, Kuder–Richardson Formula 20 (KR-20)/Cronbach’s alpha was calculated for the dichotomous item scores, and ordinal omega was estimated using tetrachoric correlations.

## 3 Results

### 3.1 Demographic and clinical characteristics

Among the 375 patients, females (n = 274, 73.1%) outnumbered males, most participants were aged 18 years or older (n = 232, 61.9%), most had an educational level between high school and junior college (n = 174, 46.4%), most were unmarried (n = 295, 78.7%), relatively few had grown up in reconstituted families (n = 42, 11.2%), and most had full siblings (n = 239, 63.7%). The demographic characteristics of the sample are summarized in **Table 1**. The primary clinical diagnostic composition of the sample by DIB-R status is shown in **Table 2**. The most common primary diagnoses were depressive disorders, mixed anxiety and depressive disorder, adjustment disorders, and other mood disorders. Using the DIB-R as the reference standard, 197 participants (52.5%) were classified as DIB-R positive and 178 (47.5%) as DIB-R negative. This high DIB-R-positive proportion should be interpreted in the context of the tertiary psychiatric clinical setting and the clinically enriched nature of the sample.

**Table 1:** Demographic characteristics of the sample (n = 375).

Characteristic	n (%)
Sex	
Male	101 (26.9)
Female	274 (73.1)
Age group	
<18 years	143 (38.1)
≥18 years	232 (61.9)
Educational level	
Junior high school or below	86 (22.9)
High school to junior college	174 (46.4)
Bachelor’s degree or higher	115 (30.7)
Marital status	
Married	71 (18.9)
Unmarried	295 (78.7)
Divorced	7 (1.9)
Widowed	2 (0.5)
Grew up in a reconstituted family	
Yes	42 (11.2)
No	333 (88.8)
Sibling status	
Only child	99 (26.4)
Full siblings	239 (63.7)
Half siblings	37 (9.9)

**Table 2:** Primary clinical diagnoses of the sample by DIB-R status.

Primary clinical diagnosis	Total n = 375	DIB-R negative n = 178	DIB-R positive n = 197
Depressive disorders	91 (24.3)	28 (15.7)	63 (32.0)
Anxiety disorders	25 (6.7)	15 (8.4)	10 (5.1)
Mixed anxiety and depressive disorder	64 (17.1)	35 (19.7)	29 (14.7)
Other mood disorders	40 (10.7)	11 (6.2)	29 (14.7)
Adjustment disorders	58 (15.5)	30 (16.9)	28 (14.2)
Obsessive-compulsive disorder	24 (6.4)	13 (7.3)	11 (5.6)
Bipolar affective disorder	13 (3.5)	6 (3.4)	7 (3.6)
Somatization disorder	12 (3.2)	12 (6.7)	0 (0.0)
Adolescent emotional disorder	16 (4.3)	6 (3.4)	10 (5.1)
Schizophrenia	9 (2.4)	9 (5.1)	0 (0.0)
Social phobia	7 (1.9)	3 (1.7)	4 (2.0)
Bulimia nervosa	7 (1.9)	3 (1.7)	4 (2.0)
Diagnosis unspecified	9 (2.4)	7 (3.9)	2 (1.0)
Total	375 (100.0)	178 (100.0)	197 (100.0)

Values are presented as n (%). Percentages are column percentages. Primary diagnoses were extracted from medical records. Detailed psychiatric comorbidities were not systematically recorded; therefore, this table describes primary clinical diagnoses rather than comorbidity patterns. Because several primary diagnostic categories had small cell counts, no inferential comparison across diagnostic categories was conducted.

### 3.2 Structural validity and criterion-related validity of the Chinese MSI-BPD

#### 3.2.1 Structural validity and model comparison

**Figure 1:**
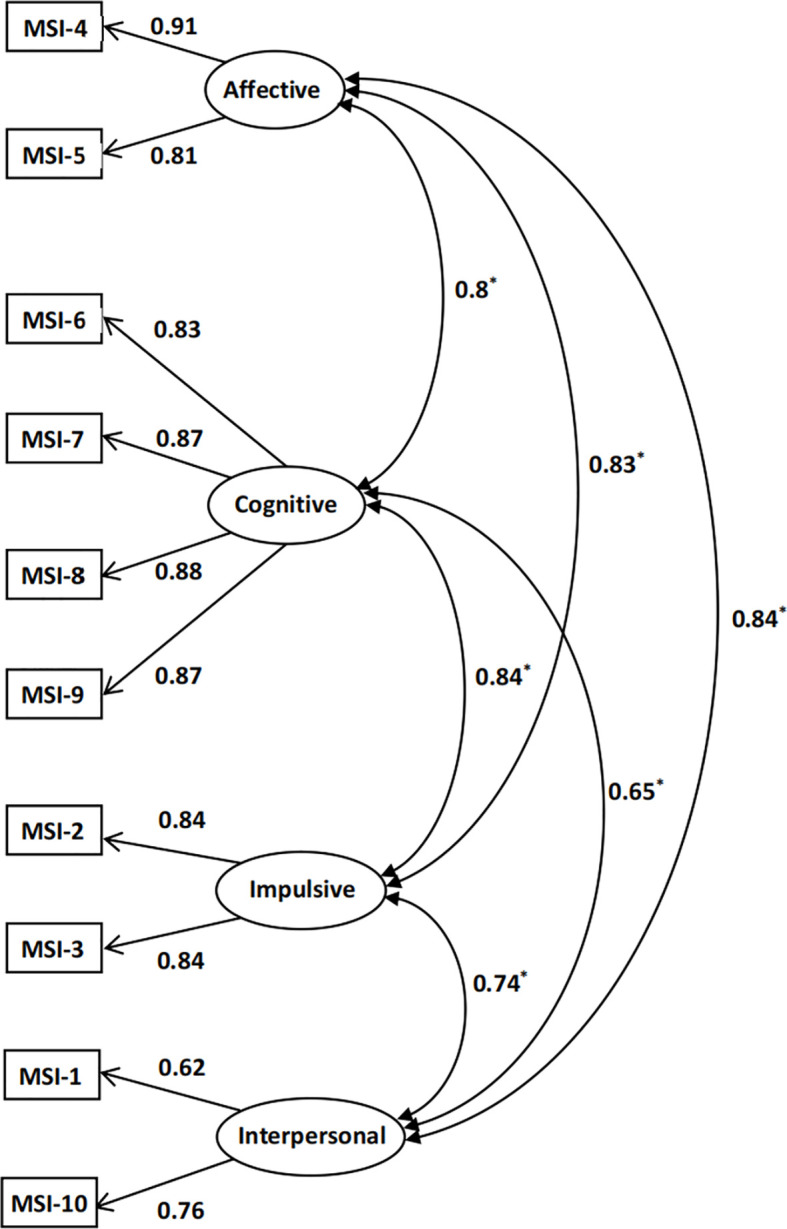
Standardized path diagram of the theory-driven four-factor model of the Chinese MSI-BPD. Single-headed arrows indicate standardized factor loadings, and double-headed arrows indicate correlations between latent factors.

The theory-driven four-factor model was compared with several theoretically plausible CFA models to evaluate the structure of the Chinese MSI-BPD. As shown in **Table 3**, the one-factor model showed acceptable but less favorable fit (χ²/df = 2.790, CFI = 0.972, TLI = 0.964, RMSEA = 0.069, 90% CI: 0.053–0.086, SRMR = 0.070), suggesting that the 10 items shared a meaningful general BPD factor. The classical four-factor model, in which MSI8 was assigned to the affective factor, showed improved fit (χ²/df = 1.954, CFI = 0.988, TLI = 0.981, RMSEA = 0.051, 90% CI: 0.031–0.070, SRMR = 0.048). The modified four-factor model reported by Chen et al. also showed acceptable conventional fit indices (χ²/df = 2.540, CFI = 0.980, TLI = 0.969, RMSEA = 0.064, 90% CI: 0.046–0.083, SRMR = 0.062). However, this model yielded a non-positive definite latent factor covariance matrix, suggesting a potentially unstable factor solution in the present sample. Therefore, although this model was included as an important literature-derived comparator, it was not retained as the preferred model. The theory-driven four-factor model, in which MSI8 was assigned to the cognitive factor, showed the most favorable fit indices among the admissible models (χ²/df = 1.409, CFI = 0.995, TLI = 0.992, RMSEA = 0.033, 90% CI: 0.000–0.055, SRMR = 0.043). The standardized factor-loading path diagram of this theory-driven four-factor model is presented in **Figure 1**. The higher-order model also showed good fit (χ²/df = 1.524, CFI = 0.993, TLI = 0.990, RMSEA = 0.037, 90% CI: 0.012–0.058, SRMR = 0.048), supporting the presence of a general BPD factor above the four first-order dimensions. These findings indicate that the theory-driven four-factor structure may provide a conceptually meaningful but provisional framework for interpretation. However, because three of the four first-order factors were defined by only two items and the higher-order model also showed good fit, the domain-level scores should be interpreted cautiously rather than being regarded as robust independent subscales. Accordingly, the MSI-BPD total score should remain the primary screening index.

**Table 3:** Fit indices for confirmatory factor analysis models of the Chinese MSI-BPD.

Model	χ²/df	CFI	TLI	RMSEA	90% CI for RMSEA	SRMR
One-factor model	2.790	0.972	0.964	0.069	0.053–0.086	0.070
Classical four-factor model	1.954	0.988	0.981	0.051	0.031–0.070	0.048
Chen et al. modified four-factor model	2.540	0.980	0.969	0.064	0.046–0.083	0.062
Theory-driven four-factor model	1.409	0.995	0.992	0.033	0.000–0.055	0.043
Higher-order model	1.524	0.993	0.990	0.037	0.012–0.058	0.048

The classical four-factor model assigned MSI8 to the affective factor, whereas the theory-driven four-factor model assigned MSI8 to the cognitive factor. The Chen et al. modified four-factor model assigned MSI6 to the interpersonal factor, whereas the theory-driven four-factor model assigned MSI6 to the cognitive factor. The Chen et al. modified four-factor model yielded a non-positive definite latent factor covariance matrix; therefore, its fit indices should be interpreted cautiously

#### 3.2.2 Convergent validity

For the theory-driven four-factor model, the standardized factor loadings ranged from 0.622 to 0.911, and all were above 0.50 (p < 0.001), indicating that each item adequately reflected its intended latent factor. The affective, cognitive, and impulsive dimensions had AVE values of 0.739, 0.741, and 0.701, respectively, and CR values of 0.849, 0.920, and 0.824, respectively, all of which met the commonly recommended thresholds of AVE > 0.50 and CR > 0.70. These findings support good convergent validity for these three dimensions. In contrast, the interpersonal dimension had an AVE = 0.482 and a CR = 0.649, slightly below conventional thresholds, suggesting relatively weak convergent validity. This may be related to the small number of items related to this factor, the heterogeneity of symptoms in clinical samples, and the high co-occurrence of BPD symptoms (28, 39–40). Overall, convergent validity was acceptable to good for the affective, cognitive, and impulsive dimensions, whereas the interpersonal dimension showed weaker convergent validity and should be interpreted cautiously (**Table 4**).

**Table 4:** Standardized factor loadings, AVE, and CR of the Chinese MSI-BPD factors.

Factor	Observed items	Standardized factor loadings	AVE	CR
Affective	MSI4	0.911	0.739	0.849
	MSI5	0.805		
Cognitive	MSI6	0.827	0.741	0.920
	MSI7	0.867		
	MSI8	0.876		
	MSI9	0.872		
Impulsive	MSI2	0.835	0.701	0.824
	MSI3	0.839		
Interpersonal	MSI1	0.622	0.482	0.649
	MSI10	0.759		

#### 3.2.3 Discriminant validity

Discriminant validity of the theory-driven four-factor model was assessed using the Fornell–Larcker criterion. The square roots of the AVE for the affective, cognitive, and impulsive dimensions were 0.859, 0.861, and 0.837, respectively, and were generally greater than their correlations with most other factors, suggesting acceptable discriminant validity for these dimensions. The square root of the AVE for the interpersonal dimension was 0.694 and was lower than its correlations with the affective and impulsive dimensions, indicating partial overlap with other domains. Taken together, discriminant validity was supported for the affective, cognitive, and impulsive dimensions, whereas the interpersonal dimension showed weaker distinctiveness and should be interpreted cautiously (**Table 5**).

**Table 5:** Discriminant validity of Chinese MSI-BPD factors: correlation matrix and square root of AVE.

Factor	Affective	Cognitive	Impulsive	Interpersonal
Affective	**0.859**			
Cognitive	0.800	**0.861**		
Impulsive	0.827	0.842	**0.837**	
Interpersonal	0.835	0.651	0.740	**0.694**

Bolded values on the diagonal are the square roots of the average variance extracted (√AVE).

#### 3.2.4 Criterion-related validity

To examine criterion-related validity, the Chinese MSI-BPD total score and the domain-level scores derived from the theory-driven four-factor structure were correlated with the DIB-R total score and corresponding DIB-R dimension scores. Pearson correlation analyses revealed that Chinese MSI-BPD domain-level scores were positively correlated with the corresponding DIB-R dimensions, with correlation coefficients ranging from 0.512 to 0.653 (all p < 0.01). The impulsive dimension showed the strongest association (r = 0.653), followed by the cognitive dimension (r = 0.637). The affective (r = 0.579) and interpersonal (r = 0.512) dimensions also showed moderate positive correlations. The Chinese MSI-BPD total score was strongly positively correlated with the DIB-R total score (r = 0.829, p < 0.01), indicating good criterion-related validity (**Table 6**).

**Table 6:** Correlations of Chinese MSI-BPD factor and total scores with corresponding criterion factor and total scores.

Factor	Mean Chinese MSI-BPD score, M ± SD	Mean DIB-R score, M ± SD	*R*	*P*
Affective	1.44 ± 0.76	1.49 ± 0.62	0.579	<0.01
Cognitive	2.58 ± 1.49	1.58 ± 0.68	0.637	<0.01
Impulsive	1.35 ± 0.80	1.54 ± 1.36	0.653	<0.01
Interpersonal	1.12 ± 0.79	2.23 ± 1.17	0.512	<0.01
Total	6.49 ± 3.03	6.83 ± 3.18	0.829	<0.01

### 3.3 Reliability of the Chinese MSI-BPD

A total of 375 participants were included in the reliability analyses, and 20 randomly selected participants completed the retest assessment after 2–3 weeks.

#### 3.3.1 Internal consistency and split-half reliability

Reliability was examined for both the MSI-BPD total score and the domain-level scores derived from the theory-driven four-factor model. The Chinese MSI-BPD total scale had a McDonald’s omega of 0.884 and a Guttman split-half reliability of 0.894, indicating good internal consistency and split-half reliability. Among the dimensions, the cognitive dimension showed relatively good reliability (omega = 0.796; split-half reliability = 0.838). The affective and impulsive dimensions both had omega values of 0.641 and split-half reliability coefficients of 0.641, suggesting modest reliability, which may partly reflect their two-item structure. The interpersonal dimension showed weak reliability (omega = 0.467; split-half reliability = 0.467). These findings indicate that the MSI-BPD total scale was psychometrically more robust than the domain-level scores, and that domain-level scores, especially the interpersonal dimension, should not currently be interpreted as robust independent clinical subscales (**Table 7**).

#### 3.3.2 Test–retest reliability

Twenty randomly selected participants completed the Chinese MSI-BPD twice over a 2–3-week interval. The test–retest subsample was compared with the full sample on key demographic and clinical characteristics. As shown in **Supplementary Table 1**, the retest subsample did not differ significantly from the full sample in sex, age group, DIB-R status, baseline MSI-BPD total score, or DIB-R total score. These findings suggest that the retest subsample was broadly comparable to the full sample on these key characteristics, although the small size of the retest subsample should be considered when interpreting the test–retest reliability estimate.

The correlation coefficient for the total score across the two assessments was 0.851, and the corresponding coefficients for the domain-level scores ranged from 0.653 to 0.859 (all p < 0.05), indicating acceptable to good temporal stability (**Table 7**).

**Table 7:** Reliability coefficients of Chinese MSI-BPD dimensions and total scale.

Factor	Number of items	McDonald’s ω (n = 375)	Guttman Split-Half (n = 375)	Test–retest reliability (n = 20)
Affective	2	0.641	0.641	0.695
Cognitive	4	0.796	0.838	0.859
Impulsive	2	0.641	0.641	0.802
Interpersonal	2	0.467	0.467	0.653
Total	10	0.884	0.894	0.851

Sample size estimation for test–retest reliability was conducted using G*Power 3.1. Using the Pearson correlation coefficient as the effect size index, a two-sided significance level of α = 0.05, statistical power of 1 − β = 0.90, and an expected test–retest reliability coefficient of 0.70 were specified. The minimum required sample size was therefore calculated to be 14. To improve the stability and reliability of the test–retest results and to reduce the influence of sampling error, the sample size was moderately increased, and 20 participants were ultimately included in the test–retest reliability assessment.

### 3.4 Chinese MSI-BPD screening performance

#### 3.4.1 Item- and scale-level discrimination

Item analysis was conducted using the critical ratio method. Participants in the top 27% of the total scores were assigned to the high-score group (n = 119), and those in the bottom 27% were assigned to the low-score group (n = 115). Scores on all items were significantly higher in the high-score group than in the low-score group, with mean differences ranging from 0.530 to 0.888. The critical ratio values ranged from 11.368 to 29.665, all exceeding 3.00 (p < 0.01), indicating good item discrimination. Detailed item discrimination results are shown in **Supplementary Table 2**.

At the scale level, participants were classified into DIB-R-negative (n = 178) and DIB-R-positive (n = 197) groups according to the DIB-R reference standard. Welch’s test showed that the Chinese MSI-BPD total score was significantly higher in the DIB-R-positive group than in the DIB-R-negative group (8.41 ± 1.42 vs. 4.37 ± 2.92, p < 0.001), with a large effect size (Cohen’s d = 1.76). These findings indicate that the Chinese MSI-BPD total score effectively distinguished between DIB-R-positive and DIB-R-negative patients.

#### 3.4.2 ROC analysis and optimal cutoff

**Figure 2:**
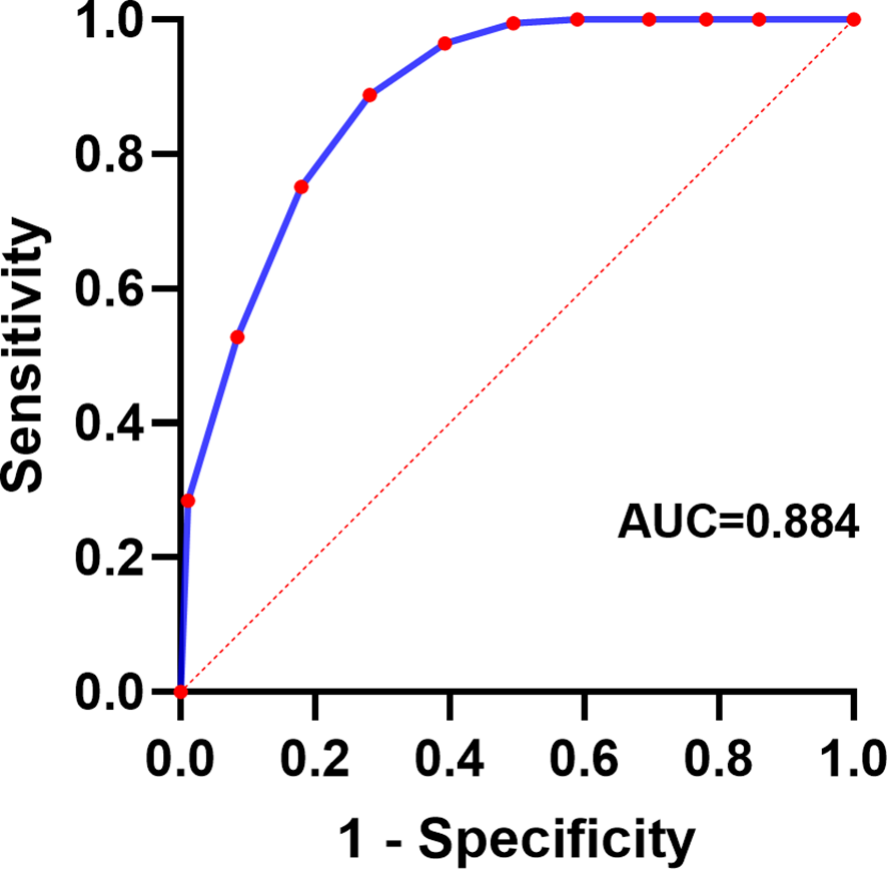
ROC curve of the Chinese MSI-BPD against the DIB-R. The ROC curve shows the screening performance of the Chinese MSI-BPD using the DIB-R as the reference criterion.

ROC analysis using the DIB-R as the reference criterion revealed that the optimal cutoff score for the Chinese MSI-BPD was 6.5 according to the maximum Youden index (0.607). Because MSI-BPD total scores are integer values, a threshold of ≥7 was considered the clinically practical cutoff in the present sample. The AUC was 0.884 (95% CI: 0.850–0.917, p < 0.001), indicating good discrimination (**Figure 2**).

#### 3.4.3 Screening performance and agreement with the DIB-R

At the cutoff of ≥7, the Chinese MSI-BPD identified 150 participants (40.0%) as negative and 225 (60.0%) as positive, whereas the DIB-R identified 178 (47.5%) as negative and 197 (52.5%) as positive. The Chinese MSI-BPD showed a sensitivity of 88.83% (95% CI: 83.67–92.51), specificity of 71.91% (95% CI: 64.90–78.00), PPV of 77.78% (95% CI: 71.90–82.72), NPV of 85.33% (95% CI: 78.79–90.11), and accuracy of 80.80% (95% CI: 76.51–84.47). The false-positive rate was 28.09% (95% CI: 22.00–35.10), and the false-negative rate was 11.17% (95% CI: 7.49–16.33). Cohen’s kappa was 0.612 (95% CI: 0.53–0.69). These results indicate moderate-to-substantial agreement between the Chinese MSI-BPD and the DIB-R and support its potential utility as an initial screening tool (**Table 8**).

**Table 8:** Screening performance and agreement between the Chinese MSI-BPD and the DIB-R at the cutoff of ≥7.

(A) Cross-classification of Chinese MSI-BPD and DIB-R results				
		DIB-R		Total
		Negative	Positive	
Chinese MSI-BPD	Negative	128	22	150
	Positive	50	175	225
	Total	178	197	375

(B) Screening performance indices		
Index	Estimate	95% CI
Sensitivity	88.83%	83.67–92.51
Specificity	71.91%	64.90–78.00
Positive predictive value	77.78%	71.90–82.72
Negative predictive value	85.33%	78.79–90.11
Accuracy	80.80%	76.51–84.47
False-positive rate	28.09%	22.00–35.10
False-negative rate	11.17%	7.49–16.33
Cohen’s kappa	0.612	0.530–0.690

CI, confidence interval; PPV, positive predictive value; NPV, negative predictive value. Confidence intervals for sensitivity, specificity, PPV, NPV, accuracy, false-positive rate, and false-negative rate were calculated using the Wilson method. The confidence interval for Cohen’s kappa was estimated using bootstrap resampling with 5,000 repetitions.

#### 3.4.4 Exploratory stratified reliability and ROC analyses by sex and age group

Exploratory subgroup analyses were conducted to examine whether the reliability and screening performance of the Chinese MSI-BPD were broadly consistent across sex and age groups. As shown in **Supplementary Table 3**, the Chinese MSI-BPD showed acceptable to good internal consistency across all subgroups. The KR-20/Cronbach’s alpha coefficients were 0.860 in males, 0.825 in females, 0.855 in participants aged 18 years or older, and 0.770 in participants younger than 18 years. The corresponding ordinal omega coefficients ranged from 0.895 to 0.932.

Stratified ROC analyses showed that the practical cutoff of ≥7 remained optimal in both males and females and in participants aged 18 years or older. In participants younger than 18 years, the Youden-based optimal cutoff was ≥6, which yielded high sensitivity but modest specificity. The fixed overall cutoff of ≥7 was further examined across subgroups (**Supplementary Table 4**). At this fixed cutoff, sensitivity remained high in both sex and age groups, but specificity and NPV were lower in participants younger than 18 years than in adults.

Given the exploratory nature of these subgroup analyses and the limited sample size available for estimating age-specific cutoff values, the age-specific cutoff of ≥6 in participants younger than 18 years should be interpreted cautiously. Therefore, the overall cutoff of ≥7 was retained as the clinically practical threshold in the present sample, while possible age-specific cutoff values require further validation in larger adolescent samples.

## 4 Discussion

To our knowledge, few studies have systematically evaluated the psychometric properties and screening performance of the Chinese version of the MSI-BPD in psychiatric clinical samples from mainland China. The present study examined the reliability, validity, and screening performance of the Chinese MSI-BPD in a tertiary psychiatric clinical sample and further tested a theory-driven four-factor model. Overall, the Chinese MSI-BPD total score showed good reliability, strong criterion-related validity with the DIB-R, and good screening performance. The theory-driven four-factor model showed the most favorable fit indices among the admissible models, whereas the higher-order model also showed good fit. These findings suggest that the MSI-BPD total score is the most psychometrically robust screening index, while the four-factor structure may provide a clinically useful but provisional interpretive framework.

The present findings should also be interpreted in relation to the early domestic psychometric study by Chen et al. (2011) (35), which first examined the MSI-BPD in Chinese psychiatric clinical samples. Consistent with that study, the present findings support the acceptable-to-good psychometric performance of the Chinese MSI-BPD in psychiatric clinical samples and suggest that multidimensional models provide a better representation of MSI-BPD item structure than a simple one-factor model. However, the specific item-domain allocation differed between the two studies. Chen et al. reported a modified four-factor model in which MSI6 was assigned to the interpersonal factor together with MSI1 and MSI10, whereas the present theory-driven model assigned MSI6, which assesses distrust or suspiciousness, to the cognitive factor together with MSI7, MSI8, and MSI9. When the Chen et al. modified four-factor model was tested in the present sample, it showed acceptable conventional fit indices but yielded a non-positive definite latent factor covariance matrix, suggesting that this structure may be less stable in the present data. By contrast, the present theory-driven four-factor model showed the most favorable fit indices among the admissible models. These differences may reflect variation in sample characteristics, clinical composition, reference standards, estimation methods, and the conceptual handling of suspiciousness within the MSI-BPD item set. The present study therefore complements and extends Chen et al. in several respects. Specifically, it used the DIB-R as a semi-structured reference standard, reported split-half reliability and test–retest reliability, evaluated AVE/CR-based construct validity, provided formal ROC-derived screening indices with confidence intervals, identified a clinically practical screening threshold, and conducted exploratory sex- and age-stratified analyses. Taken together, these findings extend the limited prior Chinese evidence and further support the cautious use of the Chinese MSI-BPD as a preliminary screening tool in tertiary psychiatric clinical settings in China.

The structural validity findings provide a more nuanced understanding of the Chinese MSI-BPD. The one-factor model showed acceptable but less optimal fit, indicating that the 10 items share a meaningful general BPD component. Both the classical four-factor model and the theory-driven four-factor model showed better fit than the one-factor model, suggesting that some domain-level organization is also present. The theory-driven model, in which MSI8 was assigned to the cognitive factor, showed the most favorable fit indices among the admissible models. However, the good fit of the higher-order model further indicates that a general BPD factor may underlie the four first-order domains. Therefore, the current findings support the use of the MSI-BPD total score as the primary screening index rather than the use of domain-level scores as independent clinical subscales.

The placement of MSI8, which assesses chronic feelings of emptiness, requires careful interpretation. In classical theoretical models of BPD, chronic emptiness is often considered part of affective disturbance (25,26). In the present theory-driven model, MSI8 was assigned to the cognitive factor because, within the MSI-BPD item set, it appears to reflect not only affective deprivation but also a persistent sense of internal hollowness, weakened self-representation, and instability of self-experience. This interpretation is consistent with the cross-domain nature of chronic emptiness, which may involve both emotional impoverishment and disturbances in self-continuity and internal representation (28–31). The strong loading of MSI8 on the cognitive factor in this sample supports this item assignment within the present model. Nevertheless, this finding should not be interpreted as evidence that chronic emptiness is exclusively cognitive. Rather, it suggests that, in the Chinese MSI-BPD, chronic emptiness may be psychometrically closer to items reflecting unreality, identity disturbance, and suspiciousness than to items reflecting mood instability or anger (33).

The domain-level findings also indicate that the four-factor structure should be interpreted cautiously. The affective, cognitive, and impulsive dimensions showed acceptable to good convergent validity, whereas the interpersonal dimension showed weaker convergent validity and weaker distinctiveness. The reliability findings showed a similar pattern: the total scale had good internal consistency and split-half reliability, while the interpersonal dimension showed weak internal consistency. This is not unexpected, because the MSI-BPD is a brief 10-item screening instrument and, in the theory-driven four-factor model, three of the four first-order factors were defined by only two items. Two-item factors are inherently more fragile and more sensitive to sample-specific variation (41). In addition, the moderate-to-high correlations among latent factors suggest that the domains derived from the theory-driven model were not fully independent in this clinical sample. Therefore, the domain-level scores, especially the interpersonal dimension, should be interpreted cautiously and should not currently be regarded as robust independent subscales.

The reliability results support the use of the Chinese MSI-BPD total score in psychiatric clinical settings. The total scale showed good McDonald’s omega, Guttman split-half reliability, and test–retest reliability, indicating that the total score provides relatively stable and internally consistent information. The lower reliability of several domain-level scores, particularly the interpersonal dimension, further supports the use of the total score as the primary screening index rather than the domain-level scores. In practical use, clinicians should therefore treat the MSI-BPD as a brief global screening instrument rather than as a multidimensional diagnostic scale.

The criterion-related validity findings also support the clinical relevance of the Chinese MSI-BPD. The total score showed a strong positive correlation with the DIB-R total score, indicating that MSI-BPD total scores were closely aligned with interview-based assessment of BPD features. Domain-level correlations with corresponding DIB-R dimensions were moderate, suggesting that the domain-level scores derived from the theory-driven model showed some alignment with interview-based DIB-R dimensions. However, because the domain-level scores showed weaker reliability and partial overlap, these correlations should be interpreted as supportive evidence for clinical relevance rather than as confirmation of independent subscale validity.

The screening performance analyses further support the utility of the Chinese MSI-BPD as a brief preliminary screening instrument. In the present sample, all items showed satisfactory discrimination, and ROC analysis indicated good overall discrimination and supported ≥7 as a clinically practical threshold, which is broadly consistent with previous MSI-BPD studies (19, 36). At this threshold, the scale showed high sensitivity and acceptable specificity, and Cohen’s kappa indicated moderate-to-substantial agreement with the DIB-R. These findings suggest that the Chinese MSI-BPD may be useful for identifying patients who require further structured assessment for BPD in tertiary psychiatric clinical settings.

At the same time, the screening results should be interpreted in the context of the sample. The DIB-R-positive rate in the present sample was 52.5%, indicating a clinically enriched tertiary psychiatric sample rather than a representative general psychiatric population. Because PPV and NPV are prevalence-dependent, the predictive values observed in this study may not directly generalize to clinical settings with lower base rates of BPD (42). In lower-prevalence settings, the PPV would be expected to decrease, meaning that a larger proportion of positive screening results would require further diagnostic confirmation. Conversely, the NPV would generally increase, making a negative result more useful for ruling out elevated BPD risk. Therefore, the Chinese MSI-BPD should be understood as a preliminary screening tool, and positive screening results should be followed by structured or semi-structured clinical assessment rather than being interpreted as diagnostic confirmation.

The exploratory stratified analyses provide additional but preliminary information about subgroup performance. Reliability was acceptable to good across sex and age subgroups. Stratified ROC analyses showed that the practical cutoff of ≥7 remained optimal in both males and females and in participants aged 18 years or older. Among participants younger than 18 years, the optimal cutoff was ≥6, which yielded high sensitivity but modest specificity. When the fixed overall cutoff of ≥7 was applied, sensitivity remained high in the younger subgroup, but specificity and NPV were lower than those observed in adults. These findings suggest that the overall cutoff of ≥7 is clinically practical for the total sample and appears stable across sex groups and adult participants. However, possible age-specific cutoffs, particularly in adolescent samples, require further validation before clinical recommendation.

The present findings have practical implications for psychiatric clinical settings in China. BPD remains under-recognized in many routine clinical contexts, partly because its symptoms overlap with mood, anxiety, trauma-related, psychotic-spectrum, and other psychiatric presentations. A brief self-report screening tool such as the Chinese MSI-BPD may help clinicians identify patients who warrant further structured assessment (17, 19, 36). However, because the MSI-BPD is not a diagnostic instrument, it should be used as the first step in a stepped assessment process. In clinical practice, a positive MSI-BPD result should prompt further clinical interview, review of longitudinal symptom patterns, assessment of functional impairment, and differential diagnosis rather than immediate diagnostic labeling.

## 5 Limitations and future directions

Several limitations should be acknowledged. First, this study recruited participants from only one tertiary psychiatric hospital in Zhuhai, Guangdong Province, using convenience sampling among clinically available outpatients and inpatients rather than consecutive, multicenter, or population-based sampling. Therefore, the sample should be understood as a clinically enriched tertiary psychiatric sample rather than a representative general psychiatric population. Accordingly, the findings cannot be generalized to general psychiatric outpatient settings, primary mental health services, or community populations. In addition, the DIB-R-positive rate was high in the present sample. Although this enriched case composition is useful for evaluating screening performance in a clinically relevant setting, it may limit the generalizability of prevalence-dependent indices such as PPV and NPV. The cutoff and predictive values observed in this study should therefore be validated in broader psychiatric populations with more representative base rates of BPD. The sample was also skewed toward female participants, and although exploratory sex-stratified analyses showed acceptable-to-good reliability and good ROC performance in both males and females, the generalizability of the findings to male patients should still be interpreted cautiously.

Second, the theory-driven four-factor structure should be interpreted cautiously. The MSI-BPD is a brief 10-item screening instrument, and three of the four first-order factors were represented by only two items. This limits the reliability, stability, and interpretability of the domain-level scores. The interpersonal dimension showed particularly weak internal consistency and weaker convergent and discriminant validity. Therefore, the four-factor model should be regarded as a clinically useful but provisional interpretive framework rather than evidence that the MSI-BPD provides robust independent subscale scores. The MSI-BPD total score should remain the primary screening index.

Third, although the higher-order model supported the presence of a general BPD factor, the stability of the factor structure requires further testing in independent samples. Future studies should examine whether the theory-driven four-factor model and the higher-order structure can be replicated in larger samples, different clinical settings, adolescent-focused samples, and community populations. Measurement invariance across sex and age groups should also be examined in future research with larger and more balanced subgroup samples. Additionally, the tentative optimal cutoff of ≥6 for participants younger than 18 years remains exploratory and requires validation in larger independent adolescent samples before clinical adoption.

Fourth, test–retest reliability was assessed in only 20 participants, which may limit the precision and stability of the temporal reliability estimate. Although the retest subsample did not differ significantly from the full sample in sex, age group, DIB-R status, baseline MSI-BPD total score, or DIB-R total score, the small retest sample means that the test–retest coefficient should be interpreted cautiously. Future studies should confirm the temporal stability of the Chinese MSI-BPD in larger retest samples.

Fifth, although primary clinical diagnoses were described, detailed psychiatric comorbidities were not systematically recorded. Therefore, this study could not examine how comorbid psychiatric conditions may have influenced MSI-BPD scores, false-positive results, or screening performance. Future studies should collect more detailed diagnostic and comorbidity information, particularly regarding mood disorders, anxiety disorders, trauma-related disorders, substance use disorders, eating disorders, and psychotic disorders.

Finally, although all DIB-R interviewers received standardized training and were blinded to the Chinese MSI-BPD scores, formal inter-rater reliability for the DIB-R reference assessment was not quantified. Therefore, potential variability among interviewers cannot be fully excluded. Future studies should include independent double-rating or audio/video-based co-rating procedures and report formal DIB-R inter-rater reliability indices. In addition, because the Chinese MSI-BPD is a self-report screening instrument, positive screening results should continue to be followed by structured or semi-structured clinical interviews when diagnostic decisions are required.

## 6 Conclusions

The Chinese MSI-BPD total score demonstrated good reliability, criterion-related validity, and screening performance in this tertiary psychiatric clinical sample. The theory-driven four-factor model showed the most favorable fit indices among the admissible models and may provide a clinically useful but provisional interpretive framework. However, the good fit of the higher-order model, the high correlations among latent domains, and the weaker performance of several two-item factors indicate that the MSI-BPD total score should remain the primary screening index. Domain-level scores, especially the interpersonal dimension, should be interpreted cautiously and should not be regarded as robust independent subscales.

A cutoff of ≥7 appears to be a clinically practical threshold for preliminary BPD screening in the present sample. However, because the sample was clinically enriched and had a high DIB-R-positive rate, the cutoff and predictive values require further validation in larger, multicenter, and less clinically enriched psychiatric samples. The Chinese MSI-BPD may be useful as a brief initial screening tool in tertiary psychiatric clinical settings in China, but positive screening results should be followed by structured clinical assessment rather than being interpreted as diagnostic confirmation. Importantly, because the Chinese MSI-BPD was validated in the present study only in a clinically enriched tertiary psychiatric sample, it should not be used as a screening or diagnostic tool in general psychiatric or community populations until dedicated validation studies in those settings are available.

## Data Availability

The raw data supporting the conclusions of this article will be made available by the authors, without undue reservation.
