# Supplementary material for: Reliability, validity, and screening performance of the Chinese version of the McLean Screening Instrument for Borderline Personality Disorder in a psychiatric clinical sample
Source: Front Psychiatry. 2026 Jun 24;17:1865970. doi: 10.3389/fpsyt.2026.1865970 (PMC13343225; doi:10.3389/fpsyt.2026.1865970)
Supplement: Supplementary file 1 [file Table1.docx]

Supplementary Material

Supplementary Table 1. Comparison between the test–retest subsample and the full sample

| Variable | Retest subsample (n = 20) | Full sample (n = 375) | Statistic | p |
| --- | --- | --- | --- | --- |
| Female sex, n (%) | 11 (55.0) | 274 (73.1) | — | 0.121 |
| Age <18 years, n (%) | 10 (50.0) | 143 (38.1) | — | 0.348 |
| DIB-R positive, n (%) | 10 (50.0) | 197 (52.5) | — | 0.823 |
| MSI-BPD total score, mean ± SD | 5.95 ± 2.33 | 6.49 ± 3.03 | U = 3035.500 | 0.148 |
| DIB-R total score, mean ± SD | 6.95 ± 2.84 | 6.83 ± 3.18 | U = 3714.500 | 0.942 |

Note. Categorical variables were compared using Fisher’s exact tests, and continuous variables were compared using Mann–Whitney U tests.

Supplementary Table 2. Item discrimination results for the Chinese MSI-BPD

| Item | Low-score group (n = 115) | High-score group  (n = 119) | Mean difference | Critical ratio | *p* |
| --- | --- | --- | --- | --- | --- |
| MSI1 | 0.34 ± 0.475 | 0.92 ± 0.279 | 0.577 | 11.368 | < 0.01 |
| MSI2 | 0.19 ± 0.395 | 0.95 ± 0.220 | 0.758 | 18.224 | < 0.01 |
| MSI3 | 0.32 ± 0.469 | 0.98 ± 0.129 | 0.661 | 14.811 | < 0.01 |
| MSI4 | 0.47 ± 0.501 | 1.00 ± 0.000 | 0.53 | 11.545 | < 0.01 |
| MSI5 | 0.24 ± 0.431 | 0.96 ± 0.201 | 0.715 | 16.331 | < 0.01 |
| MSI6 | 0.30 ± 0.458 | 0.97 ± 0.157 | 0.679 | 15.26 | < 0.01 |
| MSI7 | 0.08 ± 0.270 | 0.97 ± 0.181 | 0.888 | 29.665 | < 0.01 |
| MSI8 | 0.37 ± 0.484 | 1.00 ± 0.000 | 0.635 | 14.32 | < 0.01 |
| MSI9 | 0.09 ± 0.283 | 0.94 ± 0.236 | 0.854 | 25.097 | < 0.01 |
| MSI10 | 0.16 ± 0.365 | 0.80 ± 0.403 | 0.642 | 12.757 | < 0.01 |

Supplementary Table 3. Stratified reliability and ROC performance of the Chinese MSI-BPD by sex and age group

| **Grouping variable** | **Subgroup** | **N** | **KR-20** | **Ordinal omega** | **AUC** | **AUC**  **95% CI** | **Optimal cutoff** | **Sensitivity, 95% CI** | **Specificity, 95% CI** | **PPV, 95% CI** | **NPV, 95% CI** | **Accuracy, 95% CI** |
| --- | --- | --- | --- | --- | --- | --- | --- | --- | --- | --- | --- | --- |
| Sex | Male | 101 | 0.860 | 0.932 | 0.924 | 0.878–0.971 | ≥7 | 0.875 (0.739–0.945) | 0.803 (0.687–0.884) | 0.745 (0.605–0.847) | 0.907 (0.801–0.960) | 0.832 (0.747–0.892) |
| Sex | Female | 274 | 0.825 | 0.920 | 0.860 | 0.816–0.904 | ≥7 | 0.892 (0.833–0.931) | 0.675 (0.586–0.753) | 0.787 (0.721–0.840) | 0.823 (0.735–0.886) | 0.799 (0.748–0.842) |
| Age group | ≥18 years | 232 | 0.855 | 0.931 | 0.900 | 0.862–0.937 | ≥7 | 0.882 (0.801–0.933) | 0.763 (0.685–0.826) | 0.713 (0.625–0.788) | 0.906 (0.839–0.947) | 0.810 (0.755–0.856) |
| Age group | <18 years | 143 | 0.770 | 0.895 | 0.824 | 0.748–0.901 | ≥6 | 0.971 (0.919–0.990) | 0.513 (0.362–0.661) | 0.842 (0.766–0.896) | 0.870 (0.679–0.955) | 0.846 (0.778–0.896) |

Note. Optimal cutoffs were determined by the maximum Youden index within each subgroup. Confidence intervals for sensitivity, specificity, positive predictive value, negative predictive value, and accuracy were estimated using the Wilson method. These subgroup analyses were exploratory.

Supplementary Table 4. Screening performance of the fixed overall cutoff of ≥7 by sex and age group

| **Grouping variable** | **Subgroup** | **N** | **Cutoff** | **Sensitivity, 95% CI** | **Specificity, 95% CI** | **PPV, 95% CI** | **NPV, 95% CI** | **Accuracy, 95% CI** |
| --- | --- | --- | --- | --- | --- | --- | --- | --- |
| Sex | Male | 101 | ≥7 | 0.875 (0.739–0.945) | 0.803 (0.687–0.884) | 0.745 (0.605–0.847) | 0.907 (0.801–0.960) | 0.832 (0.747–0.892) |
| Sex | Female | 274 | ≥7 | 0.892 (0.833–0.931) | 0.675 (0.586–0.753) | 0.787 (0.721–0.840) | 0.823 (0.735–0.886) | 0.799 (0.748–0.842) |
| Age group | ≥18 years | 232 | ≥7 | 0.882 (0.801–0.933) | 0.763 (0.685–0.826) | 0.713 (0.625–0.788) | 0.906 (0.839–0.947) | 0.810 (0.755–0.856) |
| Age group | <18 years | 143 | ≥7 | 0.894 (0.820–0.940) | 0.564 (0.410–0.707) | 0.845 (0.766–0.901) | 0.667 (0.496–0.802) | 0.804 (0.732–0.861) |

Note. The fixed cutoff analysis used the overall clinically practical threshold of ≥7. Confidence intervals were estimated using the Wilson method. These subgroup analyses were exploratory and should not be used to establish subgroup-specific clinical cutoffs.
